# Association Between Short‐Term Blood Pressure Variability and the Alzheimer's Disease Continuum

**DOI:** 10.1002/brb3.70990

**Published:** 2025-10-15

**Authors:** Xinying Zou, Qiwei Ren, Wenyi Li, Shirui Jiang, Linlin Wang, Shiyi Yang, Min Zhao, Tianlin Jiang, Huiying Zhang, Fenshuang Zheng, Jun Xu, Jiwei Jiang

**Affiliations:** ^1^ Department of Neurology, Beijing Tiantan Hospital Capital Medical University Beijing China; ^2^ China National Clinical Research Center For Neurological Diseases Beijing China; ^3^ Department of Health Management, Beijing Tiantan Hospital Capital Medical University Beijing China; ^4^ Department of Emergency Medicine Second People's Hospital of Yunnan Province Kunming China; ^5^ Department of Neurology The Second People's Hospital of Guiyang Guiyang China

**Keywords:** Alzheimer's disease, biomarker, blood pressure variability, neuropsychiatric symptoms

## Abstract

**Background:**

Emerging evidence indicates that blood pressure variability (BPV) is a modifiable vascular risk factor for Alzheimer's disease (AD). Herein, we aimed to systematically evaluate the potential role of short‐term BPV across the AD continuum.

**Methods:**

This study included 263 patients on the AD continuum from the Chinese Imaging, Biomarkers, and Lifestyle study between January 1, 2023, and December 31, 2023. 24‐h ambulatory blood pressure monitoring was performed to obtain blood pressure measurements and evaluate BPV. Partial Spearman's correlation and restricted cubic spline analysis were performed to assess the associations between BPV and neuropsychological tests, cerebrospinal fluid biomarkers, and multimodal neuroimaging measures, respectively. The mediating effects of multimodal neuroimaging measures on the association between BPV and neuropsychiatric symptoms (NPS) were analyzed.

**Results:**

The elevated standard deviation (SD) and average real variability of nightly diastolic blood pressure (DBP) were correlated with higher Neuropsychiatric Inventory (NPI) scores (*r* = 0.19, *p* = 0.034; *r* = 0.22, *p* = 0.013). The increased SD of nightly systolic blood pressure (SBP) was correlated with increased Hamilton Anxiety Scale (HAMA) scores and total tau levels (*r* = 0.20, *p* = 0.026; *r* = 0.17, *p* = 0.027), and elevated coefficient of variation (CV) of nightly SBP was correlated with higher HAMA scores and lower Aβ_42/40_ levels (*r* = 0.20, *p* = 0.026; *r* = –0.18, *p* = 0.021). The nightly variability of SBP showed an inverted U‐shaped relationship with Montreal Cognitive Assessment scores (*P* for nonlinear = 0.008; *P* for nonlinear = 0.015; *P* for nonlinear = 0.021). The left cuneus volume mediated 29.41% of the association between the CV of nightly SBP and HAMA scores, while the right medial orbitofrontal thickness mediated 35.44% of the association between the CV of nightly DBP and NPI scores.

**Conclusion:**

This study suggests that short‐term BPV may play a role in the AD continuum. These findings provide evidence of a vascular pathway to AD, as well as a potential and accessible intervention target for patients on the AD continuum.

## Graphical abstract

Short‐Term blood pressure variability (BPV), particularly nightly BPV, is associated with cognition, neuropsychiatric symptoms (NPS), multiple cerebrospinal fluid biomarkers, and multimodal neuroimaging measures across the Alzheimer's disease (AD) continuum. The correlation between BPV and NPS was partly mediated by the left cuneus volume and right medial orbitofrontal thickness.

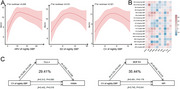

## 1 Introduction

The pathophysiology of Alzheimer's disease (AD) is intricate and complex, and increasing evidence has suggested that neurovascular dysfunction contributes to the abnormal accumulation of amyloid beta (A*β*), a hallmark feature of AD (Fisher et al. 2022). Age‐related vascular changes accompany, or even precede, the development of AD pathology, raising the possibility that they may play a pathogenic role (Cortes‐Canteli and Iadecola 2020). A recent study identified hypertension as a particularly important and modifiable vascular risk factor for AD (Livingston et al. 2024). Hypertension deteriorates the cerebral microcirculation's structural and functional integrity, impacting cerebral blood flow (CBF) and aggravating amyloid pathologies (Ungvari et al. 2021). A long‐term follow‐up study found that sustained hypertension from mid‐life to late‐life, mid‐life hypertension, and late‐life hypotension were associated with an increased risk of dementia (Walker et al. 2019). A cohort study reported that lower blood pressure was associated with higher risk of cognitive impairment (Mossello et al. 2015); however, a randomized controlled trial of 9361 cognitively normal participants demonstrated that intensive antihypertensive therapy significantly reduced the risk of dementia (SPRINT MIND Investigators for the SPRINT Research Group et al. 2019). These findings underscore the high heterogeneity in the effects of blood pressure level on patients with AD.

Blood pressure variability (BPV), defined as fluctuations in blood pressure, has been increasingly recognized as a key contributor to neurodegenerative diseases and a biomarker of aging, driven by arterial stiffness, autonomic dysfunction, and arterial aging (Sun 2023; Bencivenga et al. 2022). Elevated visit‐to‐visit BPV has been linked to AD progression in patients with mild‐to‐moderate AD, and increased systolic BPV has shown a clinically significant association with greater cognitive dysfunction among the patients with MCI or normal cognition (de Heus et al. 2019; Epstein et al. 2013). Additionally, a prospective cohort research indicated that increased visit‐to‐visit BPV was associated with lower cerebrospinal fluid (CSF) Aβ levels and higher phosphorylated tau (p‐Tau) and total tau (t‐Tau) levels (Sible et al. 2022). However, previous studies have mainly focused on long‐term BPV, such as day‐to‐day or visit‐to‐visit BPV. Day‐to‐day BPV was typically assessed using home blood pressure monitoring, which was prone to measurement errors caused by irregular monitoring, medication, or changes in comorbidities (Haverkamp et al. 2022). Visit‐to‐visit BPV measures were typically derived from longitudinal studies, and the non‐standardized blood pressure measurements, varying numbers and intervals of blood pressure measurements, and discontinuation or dose adjustment of anti‐hypertensive therapy introduced potential confounding effects (Sible et al. 2022). BPV was typically evaluated in the daytime rather than at night or throughout the day; thus, it neglected the time‐specific effects of blood pressure.

More importantly, increased BPV was found to be an independent predictor for developing AD dementia (Yoo et al. 2020). However, another study involving patients with mild‐to‐moderate AD found no predictive value of visit‐to‐visit BPV for cognitive decline (O'Caoimh et al. 2019). The association of BPV with cognition remains contradictory. Additionally, while a relationship between BPV and depressive symptoms has been reported in cognitively unimpaired individuals, no such association has been found in those with the AD continuum (Sible et al. 2022). Recent evidence has indicated that increased BPV is associated with atrophy of the temporal lobe gray matter; however, the mechanism by which BPV contributes to the AD continuum remains unclear (Yu et al. 2022). Thus, in this study, we aimed to comprehensively investigate the association of BPV, measured using 24‐h ambulatory blood pressure monitoring (ABPM), with cognition, neuropsychiatric symptoms (NPS), CSF biomarkers, and multimodal neuroimaging measures. Additionally, we sought to further explore the potential mechanism underlying this association.

## 2 Materials and Methods

### 2.1 Study Population

**FIGURE 1:**
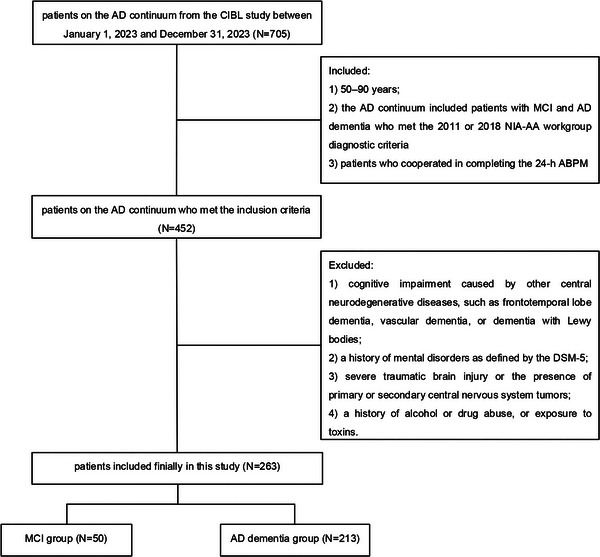
**A flowchart of the inclusion and exclusion criteria. Abbreviations**: ABPM, ambulatory blood pressure monitoring; AD, Alzheimer's disease; CIBL study, Chinese Imaging, Biomarkers, and Lifestyle study; NIA‐AA, National Institute on Aging and Alzheimer's Association; DSM‐5, Diagnostic and Statistical Manual of Mental Disorders‐5; MCI, mild cognitive impairment.

In this cross‐sectional study, data from patients on the AD continuum were obtained from the Chinese Imaging, Biomarkers, and Lifestyle (CIBL) study between January 1, 2023, and December 31, 2023. Detailed descriptions of the CIBL study design and methods have been provided in the previous study (Jiang et al. 2023). The inclusion criteria were as follows: (1) patients aged 50–90 years; (2) the AD continuum included patients with MCI and AD dementia who met the 2011 or 2018 National Institute on Aging Alzheimer's Association (NIA‐AA) workgroup diagnostic criteria (McKhann et al. 2011; Jack et al. 2018); and (3) patients who cooperated in completing the 24‐h ABPM. The exclusion criteria were as follows: (1) cognitive impairment caused by other central neurodegenerative diseases, such as frontotemporal lobe dementia, vascular dementia, or dementia with Lewy bodies; (2) a history of mental disorders as defined by the Diagnostic and Statistical Manual of Mental Disorders‐5 (American Psychiatric Association 2013); (3) severe traumatic brain injury or the presence of primary or secondary central nervous system tumors; and (4) a history of alcohol or drug abuse or exposure to toxins (Figure 1).

The CIBL study was approved by the Institutional Ethical Review Board of Capital Medical University, Beijing Tiantan Hospital (No. KY‐2021‐028‐01). Written informed consent was obtained from all participants or their caregivers.

### 2.2 Clinical Data Collection and BPV Assessment

Demographic data were collected, including sex, age, body mass index, years of education, and history of smoking and alcohol consumption. Clinical data included a history of hypertension, coronary heart disease (CHD), diabetes, antihypertensive drug use, and carrier status of the apolipoprotein E epsilon4 (*APOE* ε4). Personal histories of hypertension, diabetes, CHD, and antihypertensive drug use were derived from records of self‐reported physician‐diagnosed diseases.

Hypertension was defined as a confirmed office systolic blood pressure (SBP) of ≥ 140 mmHg or diastolic blood pressure (DBP) of ≥ 90 mmHg on at least three different days, or a physician‐confirmed diagnosis (McEvoy et al. 2024). The regular application of antihypertensive drugs was defined as the patient taking antihypertensive medication daily or at specified intervals, as prescribed by the physician, to ensure continuity and stability of blood pressure control.

The 24‐h ABPM was conducted using a unified device, which was set to record blood pressure readings at 30‐min intervals during the daytime and 1‐h intervals during the nighttime. Daytime was defined as lasting from 6 am to 10 pm, and nighttime was defined as lasting from 10:00 pm to 6:00 am the next day. The change in arterial blood pressure over a defined period was defined by BPV (Sheikh et al. 2023). The average real variability (ARV), standard deviation (SD), and coefficient of variation (CV) of blood pressure were used to assess BPV. The ARV was calculated using the following formula:

$$\mathrm{ARV}=\frac{\sum_{k=1}^{n-1}\left|BP_{K+1}-BP_{K}\right|}{N-1}$$

where N represents the number of valid blood pressure measurements and K denotes the measurement order. The CV was calculated as the SD divided by the mean blood pressure. Participants with at least seven valid daytime readings and three valid nighttime readings were defined as those who cooperated in completing the 24‐h ABPM.

### 2.3 Neuropsychological Tests

(1) Montreal Cognitive Assessment (MoCA) (Nasreddine et al. 2005): This test covers visuospatial and executive function, naming, memory and delayed memory, attention, language, abstraction, computation, and orientation, with lower scores indicating more severe cognitive impairment. (2) Neuropsychiatric Inventory (NPI) (Cummings et al. 1994): Caregivers assessed the presence of 12 neuropsychiatric disorders (delusions, hallucinations, euphoria, depression, anxiety, apathy, irritability/lability, disinhibition, agitation/aggression, aberrant motor activity, sleep disorders, and eating or appetite disorders) and their frequency and severity in the last month, with higher scores representing more severe NPS. (3) Hamilton Depression Scale (HAMD) (Hamilton 1967): The scale covers the components of anxiety, somatization, weight loss, cognitive impairment, blockages, sleep, and disorders, with higher scores representing increased depression. (4) Hamilton Anxiety Scale (HAMA) (HAMILTON 1959): The scale covers somatic anxiety and psychic anxiety, with higher scores representing more anxiety. (5) Pittsburgh Sleep Quality Index (PSQI) (Buysse et al. 1989): The scale covers subjective sleep quality, sleep latency, sleep duration, habitual sleep efficiency, sleep disturbances, use of sleeping medication, and daytime dysfunction, with higher scores representing worse sleep.

Neuropsychological tests were assessed independently by two trained neurologists. In cases of conflicting results, a senior chief physician determined the results.

### 2.4 CSF Biomarker Measurement

Patients underwent lumbar puncture (LP) in the fasting state from 8:00 to 12:00. The LP was usually conducted in the left lateral position, with the puncture site at the lumbar 3–4 or lumbar 4–5 intervertebral space, to extract 20–25 mL of CSF into sterile polypropylene test tubes. The CSF was frozen immediately on dry ice and stored at –70°C until the detection. The presence of Aβ, t‐Tau, and p‐Tau_181_ was detected using enzyme‐linked immunosorbent assays (ELISAs) (Beijing Hightrust Diagnostics, Co., Ltd., China). A*β*_42_ positivity was defined as A*β*_42_ < 650 pg/ml or an A*β*_42/40_ ratio ≤ 0.064, as reported in a previous study (Jiang et al. 2024).

### 2.5 Brain Multimodal Imaging Acquisition and Processing

Patients underwent the imaging acquisition using a 3.0‐T magnetic resonance scanner to perform structural three‐dimensional T1‐weighted imaging (3D‐T1WI) and seven‐delay pseudo‐continuous arterial spin labeling (pCASL) (SIGNA Premier; GE Healthcare, Milwaukee, WI, USA). The parameters of 3D‐T1WI of the inversion recovery gradient recalled echo sequence were as follows: repetition time = 7.3 ms; echo time = 3.0 ms; inversion time = 450 ms; flip angle = 12°; field of view = 256 mm × 256 mm; acquisition matrix = 256 × 256; slice thickness = 1.0 mm; and 48 axial slices. We used FreeSurfer (version 6.0, http://surfer.nmr.mgh.harvard.edu/) to quantify the specific brain area volume and cortical thickness. Using corrected arterial transit time values based on the third edition of the automated anatomical labeling atlas, the regional corrected CBF (cCBF) was extracted from the preprocessed pCASL image. The brain regions of interest (ROIs) included regions involved in both autonomic regulation and emotion (Kitamura et al. 2020), including the thalamus, temporal region and its subregions (hippocampus and middle temporal gyrus), amygdala, cuneus, anterior cingulate, insula, and medial orbitofrontal cortex.

### 2.6 Statistical Analysis

The mean ± SD was used to describe continuous variables that adhered to the normal distribution, and the median and interquartile range were used to describe characteristics that deviated from normality. Category variables were expressed as frequencies or percentages. Clinical characteristics, CSF biomarkers, and BPV were compared between the MCI and AD dementia groups based on independent *t*‐tests, the Mann–Whitney *U* test, and the chi‐square or Fisher's exact test. Partial Spearman's correlation was performed to evaluate the correlation between BPV and neuropsychological tests, CSF biomarkers, and multimodal neuroimaging measures. Sex, age, years of education, and history of hypertension and CHD were adjusted to assess this association. Restricted cubic spline (RCS) analysis was conducted to explore potential nonlinear relationships between BPV and neuropsychological tests or CSF biomarkers, with four knots positioned at the 5th, 35th, 65th, and 95th percentiles. Simple mediation models were conducted using bootstrapping with 5000 iterations. This established three pathways to explain the effects of BPV data on neuropsychological tests. In this framework, the total effect (TE) included natural direct effect (NDE) and natural indirect effect (NIE), and the mediation effect was calculated as a percentage (NIE/TE × 100) (Serang and Jacobucci 2020).

All statistical analyses were performed using SPSS version 29.0 (SPSS Inc., Chicago, IL, USA) and R software version 4.2.1 (R Foundation for Statistical Computing, Vienna, Austria). Two‐sided *p*‐values < 0.05 were considered statistically significant.

## 3 Results

### 3.1 Clinical Characteristics, CSF Biomarkers, and BPV of all Participants

A total of 263 participants (55.89% female) across the AD continuum from the CIBL study were enrolled in this study, with 50 and 213 patients in the MCI and AD dementia groups, respectively. Among all 263 patients across the AD continuum, 184 underwent LP and were tested for CSF biomarkers, while 113 completed neuroimaging (113 T1WI and 35 pCASL). The average age was 66.38 ± 9.46 years in this study. Compared with the MCI group, the AD dementia group had fewer years of education (*Z* = –2.87, *p* = 0.004), lower MoCA scores (*Z* = –9.73, *p* < 0.001), higher NPI scores (*Z* = –2.43, *p* = 0.015), and lower Aβ_42_ levels (*t* = –2.41, *p* = 0.017) (Tables 1, 2).

**TABLE 1:** Clinical characteristics and CSF biomarkers of all participants.

Variable	Total (*n* = 263)	MCI (*n* = 50)	AD dementia (*n* = 213)	*t/χ^2^/Z*	*p*
Age (years, × ± s)	66.38 ± 9.46	65.30 ± 7.99	66.60 ± 9.78	0.90	0.370
Sex (female, %)	147 (55.89)	30 (60.00)	117 (54.90)	0.42	0.516
Education [years, median (IQR)]	11.00 (9.00, 14.00)	12.00 (9.00, 16.00)	9.00 (9.00, 12.00)	−2.87	0.004**
BMI (kg/m2, × ± s)	23.60 ± 3.41	24.43 ± 3.66	23.41 ± 3.34	−1.93	0.055
Hypertension (yes, %)	135 (51.33)	29 (58.00)	106 (49.80)	1.10	0.294
Antihypertensive drugs (yes, %)	113 (42.97)	26 (52.00)	87 (40.80)	2.06	0.152
Diabetes (yes, %)	76 (28.90)	14 (28.00)	62 (29.10)	0.02	0.876
CHD (yes, %)	42 (15.97)	6 (12.00)	36 (16.90)	0.73	0.395
Smoking (yes, %)	58 (22.05)	12 (24.00)	46 (21.60)	0.14	0.712
Drinking (yes, %)	40 (15.21)	10 (20.00)	30 (14.10)	1.10	0.295
*APOE* ε4 carrier (yes, %)	87 (33.08)	17 (34.00)	70 (32.86)	0.07	0.792
MoCA [score, median (IQR)]	10.00 (5.00, 14.00)	17.00 (15.00, 22.00)	8.00 (4.00, 12.00)	−9.73	< 0.001**
NPI [score, median (IQR)]	2.00 (1.00, 4.00)	2.00 (0.25, 10.75)	8.00 (2.00, 19.50)	−2.43	0.015*
HAMD [score, median (IQR)]	3.00 (1.00, 5.25)	4.00 (1.50, 7.50)	3.00 (2.00, 8.00)	−0.15	0.882
HAMA [score, median (IQR)]	4.00 (2.00, 52.5)	4.00 (2.50, 7.50)	5.00 (3.00, 7.00)	−0.14	0.887
PSQI [score, median (IQR)]	3.00 (1.00, 7.00)	4.50 (1.00, 9.25)	3.00(1.00, 7.00)	−0.37	0.712
Aβ_42_ [pg/ml, × ± s]	362.35 ± 164.48	436.65 ± 197.11	351.18 ± 156.73	−2.41	0.017*
A*β*_40_ [pg/ml, median (IQR)]	6702.42 (4392.26, 9518.10)	8113.46 (5207.92, 15003.60)	6453.10 (4203.62, 9086.79)	−1.66	0.097
A*β*_42/40_ (IQR)	0.05 (0.04, 0.07)	0.05 (0.03, 0.06)	0.05 (0.04, 0.07)	−0.63	0.531
t‐Tau [pg/ml, median (IQR)]	499.03 (221.23, 860.44)	414.27 (187.53, 859.92)	509.00 (222.06, 871.04)	−0.26	0.798
p‐Tau_181_ [pg/ml, median (IQR)]	48.79 (22.29, 83.29)	47.70 (24.43, 71.73)	49.52 (22.20, 89.48)	−0.53	0.594

Abbreviations: A*β*, amyloid beta; AD, Alzheimer's disease; *APOE* ε4, apolipoprotein E epsilon4; BMI, body mass index; CHD, coronary heart disease; CSF, cerebrospinal fluid; HAMA, Hamilton Anxiety Scale; HAMD, Hamilton Depression Scale; MCI, mild cognitive impairment; MoCA, Montreal Cognitive Assessment; NPI, Neuropsychiatric Inventory; p‐Tau, phosphorylated tau; PSQI, Pittsburgh Sleep Quality Index; t‐Tau, total tau. **p < 0.01; *p < 0.05.

**TABLE 2:** Mean blood pressure and BPV of all participants.

Variable	Total (*n* = 263)	MCI (*n* = 50)	AD dementia (*n* = 213)	*t/χ^2^/Z*	*p*
Mean whole day SBP [mmHg, median (IQR)]	119.00 (112.00, 128.00)	130.50 (117.00, 143.90)	119.00 (112.00, 128.00)	−1.31	0.896
Mean whole day DBP [mmHg, × ± s]	75.92 ± 9.39	76.52 ± 9.36	76.00 ± 9.42	0.25	0.800
Mean daytime SBP [mmHg, median (IQR)]	119.00 (113.00, 130.00)	119.50 (111.00, 132.25)	119.00 (113.00, 130.00)	−0.05	0.963
Mean daytime DBP [mmHg, × ± s]	76.63 ± 9.63	76.70 ± 9.38	76.62 ± 9.70	−0.05	0.963
Mean nightly SBP [mmHg, median (IQR)]	117.00 (109.00, 128.00)	118.00 (107.00, 131.25)	117.00 (109.00, 127.75)	−0.10	0.921
Mean nightly DBP [mmHg, × ± s]	74.24 ± 10.46	7.38 ± 10.73	74.35 ± 10.42	0.33	0.739
ARV of whole day SBP [mmHg, median (IQR)]	11.17 (9.53, 4.20)	11.08 (9.62, 14.23)	11.21 (9.30, 14.18)	−0.51	0.612
ARV of whole day DBP [mmHg, median (IQR)]	8.57 (7.14, 10.58)	9.18 (7.35, 11.33)	8.43 (7.05, 10.30)	−1.59	0.112
ARV of daytime SBP[mmHg, median (IQR)]	11.11 (8.88, 14.76)	11.58 (8.83, 14.81)	11.00 (8.87, 14.78)	−0.09	0.928
ARV of daytime DBP[mmHg, median (IQR)]	8.29 (6.63, 10.84)	8.14 (6.69, 11.48)	8.35 (6.63, 10.35)	−0.72	0.469
ARV of nightly SBP[mmHg, median (IQR)]	10.83 (8.43, 13.83)	11.00 (8.98, 14.08)	10.83 (8.26, 13.81)	−0.87	0.384
ARV of nightly DBP [mmHg, median (IQR)]	8.71 (6.29, 1.75)	9.00 (6.36, 12.26)	8.69 (6.28, 11.69)	−0.87	0.382
SD of whole day SBP [mmHg, median (IQR)]	12.17 (9.90, 15.43)	12.05 (10.50, 15.73)	12.17 (9.83, 15.40)	−0.77	0.439
SD of whole day DBP [mmHg, median (IQR)]	9.25 (7.80, 10.90)	9.50 (8.25, 11.95)	9.15 (7.63, 10.88)	−1.40	0.162
SD of daytime SBP [mmHg, median (IQR)]	11.80 (9.60, 15.40)	11.65 (9.80, 16.28)	11.80 (9.53, 15.38)	−0.32	0.750
SD of daytime DBP [mmHg, median (IQR)]	8.80 (7.30, 10.70)	9.05 (7.38, 11.73)	8.60 (7.20, 10.68)	−1.25	0.211
SD of nightly SBP [mmHg, median (IQR)]	9.50 (7.40, 11.90)	9.50 (7.68, 13.03)	9.50 (7.30, 11.90)	−0.47	0.637
SD of nightly DBP [mmHg, × ± s]	7.89 ± 3.04	8.48 ± 3.14	7.75 ± 3.01	−1.51	0.132
CV of whole day SBP [%, median (IQR)]	10.10 (8.80, 12.70)	10.95 (9.18, 13.43)	10.10 (8.70, 12.40)	−1.33	0.183
CV of whole day DBP [%, median (IQR)]	12.30 (10.30, 14.60)	12.65 (11.00, 15.53)	12.20 (10.13, 14.38)	−1.62	0.104
CV of daytime SBP [%, median (IQR)]	9.90 (8.20, 12.50)	10.20 (8.28, 13.65)	9.80 (8.13, 12.40)	−0.58	0.561
CV of daytime DBP [%, median (IQR)]	11.40 (9.60, 14.00)	12.15 (9.88, 15.18)	11.30 (9.13, 13.80)	−1.53	0.126
CV of nightly SBP [%, median (IQR)]	8.20 (6.68, 10.00)	8.15 (6.60, 10.33)	8.20 (6.40, 9.88)	−0.53	0.593
CV of nightly DBP [%, × ± s]	10.79 ± 4.39	11.45 ± 4.10	10.63 ± 4.46	−1.18	0.238

Abbreviations: AD, Alzheimer's disease; ARV, average real variability; BPV, blood pressure variability; CV, coefficient of variation; DBP, diastolic blood pressure;MCI, mild cognitive impairment; SBP, systolic blood pressure; SD, standard deviation.

### 3.2 Linear Correlation of BPV With Neuropsychological Tests, CSF Biomarkers, and Multimodal Neuroimaging Measures Across the AD Continuum

Elevated SD of whole‐day and nightly DBP was correlated with higher NPI scores (*r* = 0.36, *p* < 0.001; *r* = 0.19, *p* = 0.034), and increased SD of nightly SBP was correlated with higher HAMD scores, HAMA scores, and t‐Tau levels (*r* = 0.26, *p* = 0.004; *r* = 0.25, *p* = 0.006; *r* = 0.17, *p* = 0.027). Elevated CV of nightly SBP was correlated with higher HAMD and HAMA scores but lower A*β*_42/40_ levels (*r* = 0.23, *p* = 0.010; *r* = 0.20, *p* = 0.026; *r* = –0.18, *p* = 0.021). Furthermore, increased ARV of whole‐day SBP was correlated with lower A*β*_42/40_ levels (*r* = –0.16, *p* = 0.042), while elevated ARV of nightly SBP was correlated with higher HAMD scores and t‐Tau levels (*r* = 0.23, *p* = 0.011; *r* = 0.19, *p* = 0.015). Additionally, increased ARV of nightly DBP was correlated with higher NPI scores (*r* = 0.22, *p* = 0.013).

**FIGURE 2:**
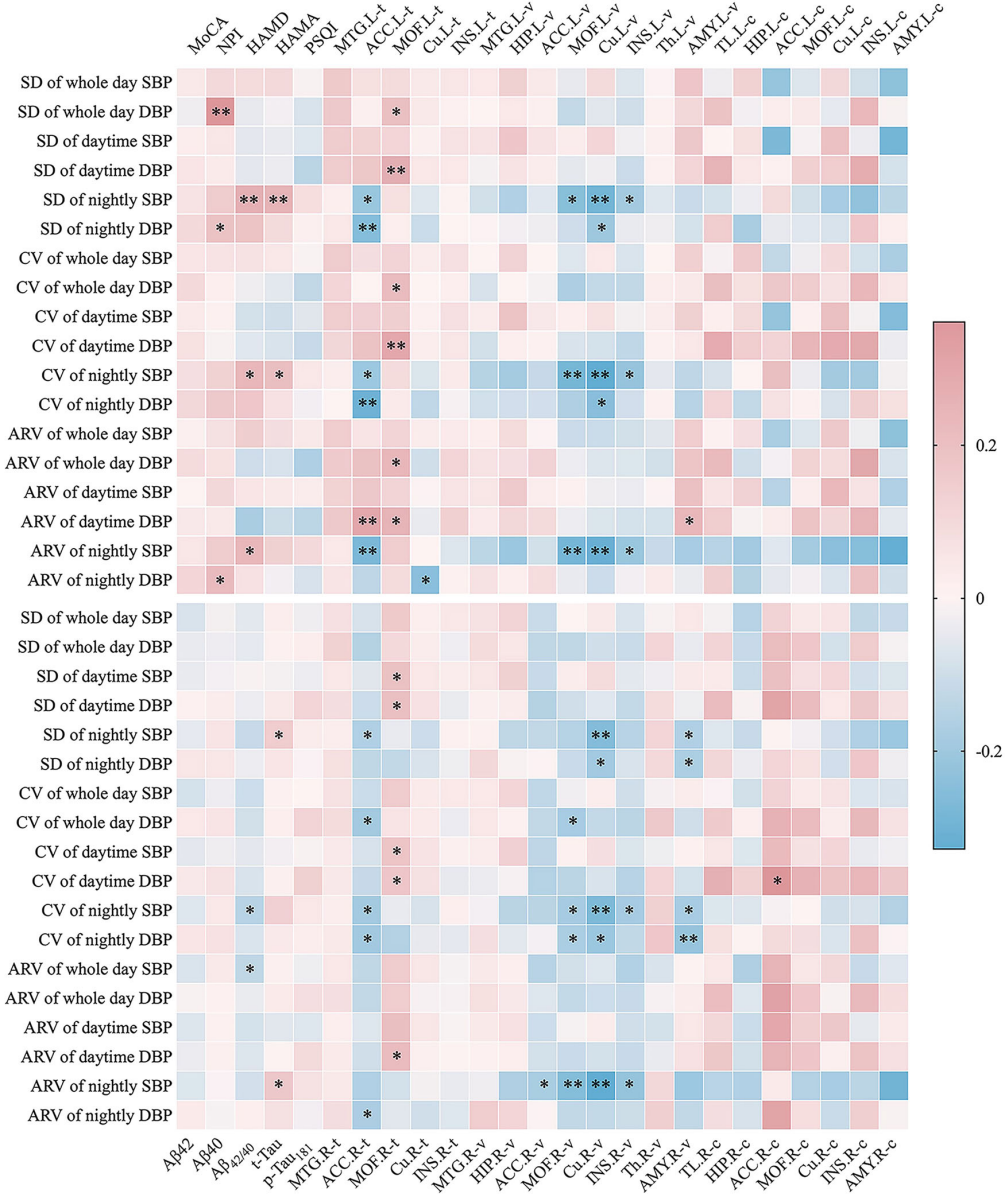
**A heatmap of partial Spearman's correlation of BPV with neuropsychological tests, CSF biomarkers, and multimodal neuroimaging measures across the AD continuum**. Age, sex, years of education, and history of hypertension and coronary heart disease were all adjusted to assess their correlations accurately. **Abbreviations**:.L, left lateral brain area;.R, right lateral brain area; −c, cerebral blood flow; −t, thickness; −v, volume; A*β*, amyloid beta; ACC, anterior cingulate cortex; AMY, amygdala; ARV, average real variability; Cu, cuneus; CV, coefficient of variation; DBP, diastolic blood pressure; HAMA, Hamilton Anxiety Scale; HAMD, Hamilton Depression Scale; HIP, hippocampus; INS, insula; MoCA, Montreal Cognitive Assessment; MOF, medial orbitofrontal cortex; MTG, middle temporal gyrus; NPI: Neuropsychiatric Inventory; p‐Tau, phosphorylated tau; PSQI: Pittsburgh Sleep Quality Index; SBP, systolic blood pressure; SD, standard deviation; t‐Tau, total tau; Th, thalamus; TL, temporal lobe. **p < 0.01; *p < 0.05.

Elevated SD and CV of nightly SBP were associated with decreased anterior cingulate cortex thickness and reduced volumes of the left medial orbitofrontal cortex, cuneus, left insula, and right amygdala. Increased SD and CV of nightly DBP were associated with reduced thickness of the left anterior cingulate cortex and decreased volumes of the cuneus and right amygdala. Additionally, elevated CV of nightly SBP was correlated with decreased volumes of the right medial orbitofrontal cortex and insula, while elevated CV of nightly DBP was associated with a decreased thickness and volume of the right medial orbitofrontal cortex. Elevated ARV of nightly SBP was correlated with a reduced thickness of the left anterior cingulate cortex and decreased volumes of the medial orbitofrontal cortex, cuneus, and insula, as well as a decreased volume of the right anterior cingulate cortex. Furthermore, increased ARV of nightly DBP was associated with a reduced thickness of the anterior cingulate cortex. Moreover, increased CV of daytime DBP was correlated with increased cCBF in the right anterior cingulate cortex. However, elevated variability of whole‐day DBP, as well as daytime SBP and DBP, was correlated with increased thickness of the medial orbitofrontal cortex, and increased ARV of daytime DBP was correlated with an increased thickness of the left anterior cingulate cortex and a larger volume of the left amygdala (all *p* < 0.05) (Figure 2).

After adjusting for covariates and mean blood pressure, the analyses continued to show that increased ARV, SD, and CV of nightly SBP were associated with reduced volumes of the medial orbitofrontal cortex, cuneus, right insula, and right amygdala. Similarly, elevated ARV, SD, and CV of nightly DBP remained significantly associated with a decreased volume of the right amygdala. Additionally, elevated ARV of whole‐day DBP and daytime SBP, along with increased SD and CV of daytime DBP, were correlated with increased cCBF in the right anterior cingulate cortex. The results of the partial Spearman's correlation analyses are summarized in the heatmaps presented in Supplementary Figure S1.

### 3.3 “U”,"J" or “S” Shaped Correlation Between BPV and Neuropsychological Tests or CSF Biomarkers Across the AD Continuum

The RCS analysis results revealed that the ARV, SD, and CV of nightly SBP exhibited an inverted U‐shaped relationship with MoCA scores (*P* for nonlinear = 0.008; *P* for nonlinear = 0.015; *P* for nonlinear = 0.021). A significant U‐shaped association was observed between the ARV of nightly SBP and NPI scores (*P* for nonlinear < 0.001), and a J‐shaped relationship was identified between the ARV of whole‐day SBP and HAMD scores (*P* for nonlinear = 0.001). Additionally, the SD of daytime SBP showed an inverted U‐shaped relationship with Aβ_42/40_ levels, while the CV of whole day SBP showed an S‐shaped relationship with Aβ_42/40_ levels (*P* for nonlinear = 0.037; *P* for nonlinear = 0.035).

**FIGURE 3:**
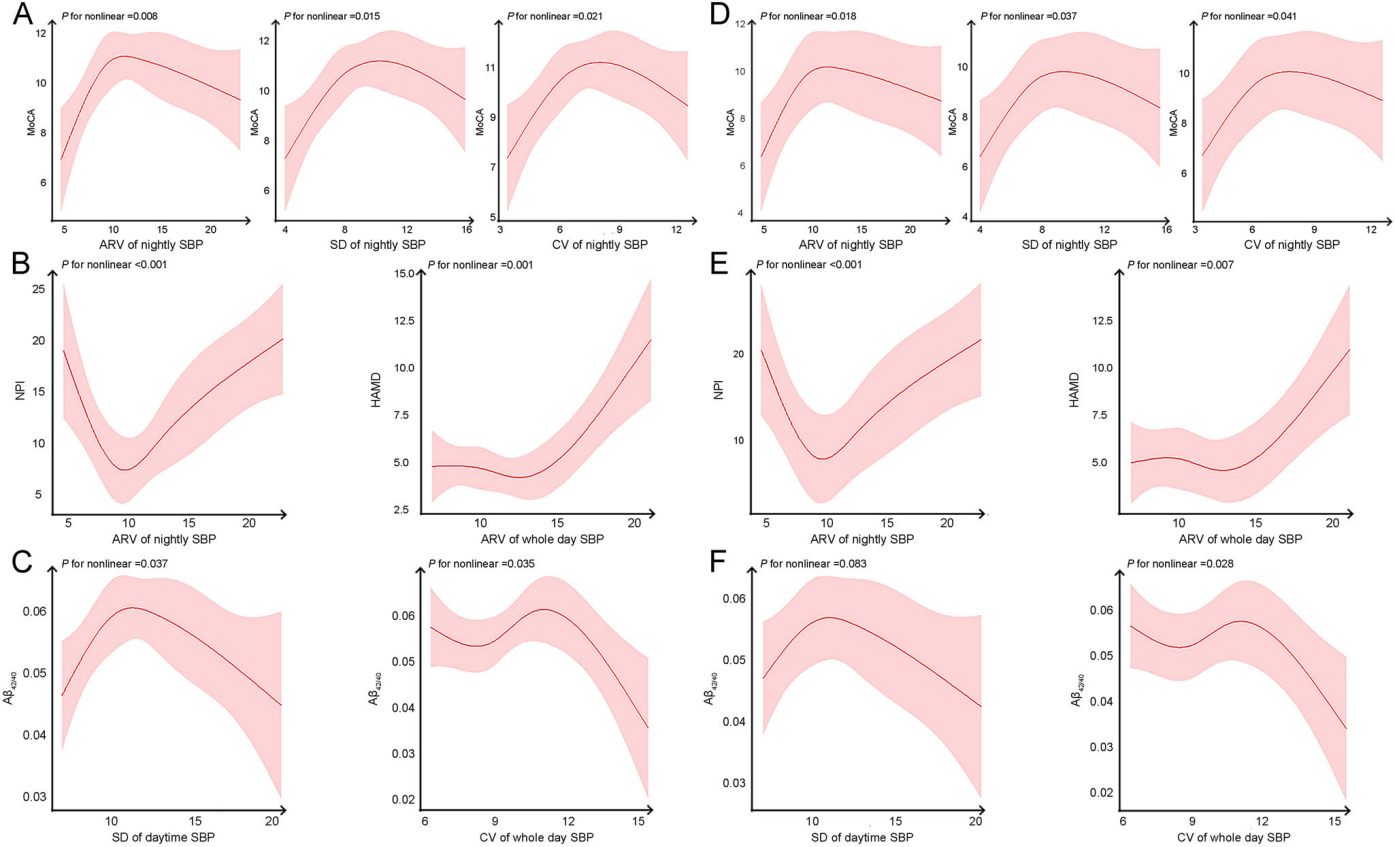
**“U”, “J” or “S” shaped correlations between BPV and neuropsychological tests or CSF biomarkers across the AD continuum. Abbreviations**: A*β*, amyloid beta; ARV, average real variability; CV, coefficient of variation; HAMD, Hamilton Depression Scale; MoCA, Montreal Cognitive Assessment; NPI, Neuropsychiatric Inventory; SBP, systolic blood pressure; SD, standard deviation. A and D show a “U” ‐shaped correlation between BPV and cognition across the AD continuum; B and E show a “U” or “J” ‐shaped correlation between BPV and NPS; and C and F show a “U” or “S” ‐shaped correlation between BPV and CSF biomarkers. D–F are adjusted for age, sex, years of education, and history of hypertension and coronary heart disease.

After adjusting for covariates, the ARV, SD, and CV of nightly SBP continued to demonstrate an inverted U‐shaped relationship with MoCA scores (*P* for nonlinear = 0.018; *P* for nonlinear = 0.037; *P* for nonlinear = 0.041). The estimated turning points for MoCA scores in relation to nightly SBP occurred at 12.4 mmHg for ARV, 10.6 mmHg for SD, and 9.2% for CV. Similarly, the U‐shaped association between the ARV of nightly SBP and NPI scores remained significant (*P* for nonlinear < 0.001), with an estimated inflection point at 9.4 mmHg. Moreover, the J‐shaped relationship between the ARV of whole‐day SBP and HAMD scores was maintained (*P* for nonlinear = 0.007), with an inflection point at 12.5 mmHg. Additionally, the relationship between the CV of whole day SBP and A*β*_42/40_ levels remained S‐shaped (*P* for nonlinear = 0.028) (Figure 3). The CV of whole‐day SBP was negatively correlated with Aβ_42/40_ levels within the range of 0–9.4 mmHg or > 11.4 mmHg, but positively correlated within the range of 9.4–11.4 mmHg.

### 3.4 Mediating Effect of ROIs on the Association Between BPV and Neuropsychological Tests Across the AD Continuum

**FIGURE 4:**
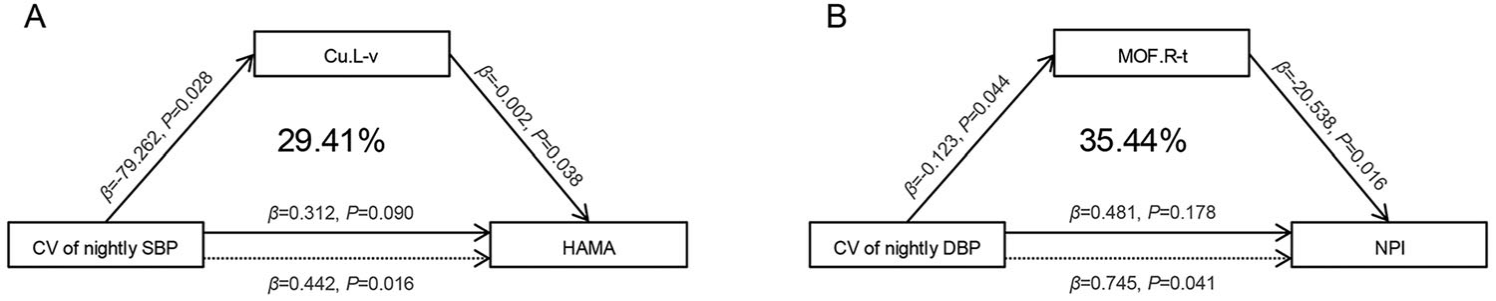
**Mediating effect of ROIs on the association between BPV and neuropsychological tests across the AD continuum**. Age, sex, years of education, and history of hypertension and coronary heart disease were all adjusted to assess the mediating effects accurately. **Abbreviations**:.L, left lateral brain area;.R, right lateral brain area; −t, thickness; −v, volume; Cu, cuneus; CV, coefficient of variation; DBP, diastolic blood pressure; HAMA, Hamilton Anxiety Scale; MOF, medial orbitofrontal cortex; NPI, Neuropsychiatric Inventory; SBP, systolic blood pressure.

The results showed that the left cuneus volume mediated 29.41% of the association between the CV of nightly SBP and HAMA scores, and the thickness of the right medial orbitofrontal mediated 35.44% of the association between the CV of nightly DBP and NPI scores (Figure 4). Detailed results of the mediating effects are shown in Supplementary Tables S1–S8.

### 3.5 Stratified Analyses of Nightly BPV in Relation to Cognition, NPS, and CSF Biomarkers

In the AD dementia subgroup, significant positive correlations were observed between the ARV, SD, and CV of nightly SBP and HAMD scores (*r* = 0.28, *p* = 0.005; *r* = 0.30, *p* = 0.003; *r* = 0.27, *p* = 0.008). Similarly, the SD and CV of nightly SBP remained positively correlated with HAMA scores (*r* = 0.29, *p* = 0.004; *r* = 0.22, *p* = 0.031). Additionally, elevated ARV of nightly DBP was correlated with higher NPI scores (*r* = 0.27, *p* = 0.005). In terms of CSF biomarkers, the CV of nightly SBP was negatively correlated with A*β*_42/40_ levels (*r* = –0.18, *p* = 0.029), while the ARV and SD of nightly SBP showed positive correlations with t‐Tau levels (*r* = 0.19, *p* = 0.021; *r* = 0.17, *p* = 0.044).

However, after adjusting for covariates, the previously observed inverted U‐shaped relationship between nightly SBP variability and MoCA scores was no longer statistically significant in the AD dementia or MCI subgroup (*P* for nonlinear > 0.05). More importantly, in the MCI subgroup, none of the above associations reached statistical significance (all *p* > 0.05).

## 4 Discussion

This study provided novel insights into the association of short‐term BPV with AD. The inverted U‐shaped relationship between nightly BPV and cognition suggested that both hypo‐ and hyper‐variability may be associated with impaired function. Elevated BPV was associated with more severe NPS across the AD continuum, potentially mediated in part by the left cuneus volume and right medial orbitofrontal thickness. The stratified analyses showed that significant associations between nightly BPV and NPS or CSF biomarkers were mainly observed in the AD dementia subgroup, with no such associations detected in the MCI subgroup, suggesting a potential stage‐dependent role of BPV.

Our results revealed that nightly variability of SBP exhibited an inverted U‐shaped relationship with MoCA scores across the AD continuum. The longitudinal study found that increased visit‐to‐visit BPV was associated with cognitive deterioration, cerebrovascular pathology, and neurofibrillary tangles in dementia‐free participants (Ma et al. 2021). Consistent with this result, our study also indicated that short‐term BPV may be an important contributing factor to cognitive decline in patients with the AD continuum. However, another longitudinal study found no correlation between visit‐to‐visit BPV and cognition in patients with mild‐to‐moderate AD (O'Caoimh et al. 2019). These discrepancies can be attributed to differences in BPV and cognition assessment methods, as well as differences in study populations (Asmuje et al. 2022). More importantly, no consensus has been reached on the appropriate BPV range. A dose–response relationship was found among cognitively unimpaired participants between long‐term BPV and cognitive decline. It suggested that the association between BPV and cognition is not simply linear, and that BPV within an appropriate range causes little damage to cognitive function (Li et al. 2021). Also, a cross‐sectional study found that both excessively high and low BPV increased the risk of death among patients with acute myocardial infarction (Liu et al. 2024). Therefore, identifying the appropriate range of BPV is critical for patients on the AD continuum.

This is the first study to report that elevated nightly BPV is associated with higher HAMA, HAMD, and NPI scores across the AD continuum. Previous studies have revealed the relationship between BPV and emotion in the anxious or depressed patients, but there have been no correlation studies in patients with AD (Virtanen et al. 2003; Nyárády et al. 2024). We further observed that the association between nightly BPV and NPS was partially mediated by the right medial orbitofrontal thickness and left cuneus volume. The orbitofrontal cortex is a key cortical region in emotion and reward, as well as being a critical region associated with NPS in patients with AD (Chen et al. 2021; Zhang et al. 2024). A recent study found that increased thickness of the left cuneus cortex was a key imaging indicator for patients with schizophrenia and bipolar disorder (Chen et al. 2021; Zhang et al. 2024). It may be a significant mood‐related brain region, which aligns with our results. Furthermore, a previous study showed that BPV modulated central neuronal activity via mechanosensitive ion channels, suggesting that BPV might be directly involved in the modulation of cognition and mood (Jammal Salameh et al. 2024).

Our results revealed that elevated variability of nightly SBP was correlated with lower A*β*_42/40_ levels and higher t‐Tau levels. Similarly, a cross‐sectional study found that elevated BPV was associated with lower levels of plasma A*β*_42_ and A*β*_42/40_ (Sible et al. 2022). Moreover, a post‐hoc analysis of the SPRINT MIND (Systolic Blood Pressure Intervention Trial Memory and Cognition in Decreased Hypertension) trial found that increased BPV was associated with higher plasma t‐Tau levels under standard blood pressure treatment, which was consistent with our results (Sible and Nation 2024). Increased BPV may have led to vascular dysfunction and greater aortic stiffness (Zhou et al. 2018), and elevated BPV was also linked to a vicious circle of arterial stiffness (Kajikawa and Higashi 2024). A previous meta‐analysis found that elevated BPV was associated with a higher burden of white matter hyperintensities. It further supported the hypothesis that elevated BPV was a marker of vascular injury (Ma et al. 2020). This finding explained how BPV could have led to dysfunction in the brain's clearance of toxic proteins (Ma et al. 2020).

We found that increased nightly BPV was associated with cerebral cortical atrophy and reduced brain volume. Similarly, the previous studies found that elevated visit‐to‐visit BPV was associated with medial temporal lobe atrophy and reduced brain volumes in cognitively normal patients (Yu et al. 2022; Sible et al. 2022). In addition, AD affects the insular cortex. Therefore, it predisposes the cardiovascular system to detrimental changes at a preclinical stage, resulting in elevated BPV (Kitamura et al. 2020). Our study also found that elevated CV of whole‐day DBP was correlated with increased cCBF in the anterior cingulate cortex. A study including patients with traumatic brain injury found that higher BPV was associated with both below‐ and above‐optimal CBF pressure, while a post‐hoc analysis of the SPRINT MIND trial found that elevated BPV was correlated with the CBF decline in the hippocampus and medial temporal regions among dementia‐free patients, which contradicted our results (Svedung Wettervik et al. 2020; Sible and Nation 2023). The underlying mechanisms may differ between short‐term and visit‐to‐visit BPV. Visit‐to‐visit BPV is mainly influenced by increased arterial stiffness, while short‐term BPV is mainly influenced by central and reflex autonomic modulation (Parati et al. 2013).

In this study, we found that the CV of whole‐day SBP showed an S‐shaped relationship with the A*β*_42/40_ level. The S‐shaped relationship between whole‐day SBP and A*β*_42/40_ levels implies that moderate BPV (9.4–11.4 mmHg) may be associated with enhanced amyloid clearance, whereas extremes may be linked to exacerbated pathology. However, previous studies have found a linear association between long‐term BPV and pathological protein deposition in older adults, with higher BPV associated with more severe A*β* and tau deposition (Sible et al. 2022; Sible et al. 2022); meanwhile, we observed a linear negative correlation between the CV of nightly SBP and Aβ_42/40_ levels, which was contradictory to this finding. One probable explanation for this difference is that blood pressure is more affected by physical activity and external stimuli during the daytime, leading to a certain bias (Sheikh et al. 2023). The finding associating increased daytime BPV with increased medial orbitofrontal thickness further underscores the inaccuracy of daytime BPV. In addition, there are differences in the parasympathetic and sympathetic nervous system activities between day and night (Sheikh et al. 2023), which could also explain this contradictory result.

This study has several strengths. It demonstrates an inverted U‐shaped association between nightly systolic BPV and cognition across the AD continuum. Moreover, it is the first study to report that elevated nightly BPV is correlated with more severe NPS across the AD continuum, which may be partially mediated by the medial orbitofrontal cortex and cuneus. However, this study also has some limitations. First, this is a single‐center cross‐sectional study, which lacks disease progression data and may introduce a certain selection bias. Due to the observational design, the observed associations between BPV and AD do not imply causality. Second, as stratified analyses reveal significant associations in the AD dementia subgroup but not in the MCI subgroup, the role of BPV in earlier stages of the disease remains unclear and warrants further investigation. Third, the inclusion criteria for valid ABPM readings may reduce the precision of short‐term BPV measurements, potentially compromising the accuracy of subsequent analyses. Fourth, the use of fixed time intervals, rather than individual sleep‐wake patterns, may limit the validity of metrics sensitive to diurnal rhythms.

## 5 Conclusion

This study comprehensively revealed that short‐term BPV, particularly nightly BPV, is associated with cognition, NPS, multiple CSF biomarkers, and multimodal neuroimaging measures across the AD continuum. The association between nightly BPV and NPS may be partially mediated by the cuneus and medial orbitofrontal cortex, which provides preliminary insights into the possible underlying mechanism linking BPV to the AD development. These findings emphasize the importance of nightly BPV in clinical management across the AD continuum, especially in patients with AD dementia, beyond blood pressure levels alone. This study also supports the evidence of a vascular pathway to AD and suggests a potential, accessible intervention target for patients across the AD continuum. Further longitudinal studies with larger sample sizes are needed to investigate the correlation of the trajectories between BPV and AD development.

## Author Contributions

XZ, QR, and JJ designed the study. XZ and QR analyzed the data and completed the manuscript. XZ, QR, JJ, WL, SJ, LW, SY, MZ, TJ, and HZ collected the data. JJ and JX modified the manuscript and approved the submission. XZ and QR contributed equally (joint first authors).

## Conflicts of Interest

The authors declare no conflicts of interest.

## Peer Review

The peer review history for this article is available at https://publons.com/publon/10.1002/brb3.70990

## Supporting information




**Supplementary Figure**: brb370990‐sup‐0001‐FigureS1.jpg

**Supplementary Tables**: brb370990‐sup‐0002‐TableS1‐S8.docx

## Data Availability

The data that support the findings of this study are available from the corresponding author upon reasonable request.
