# Correction to “Association Between Short‐Term Blood Pressure Variability and the Alzheimer's Disease Continuum”

**DOI:** 10.1002/brb3.71730

**Published:** 2026-08-26

Zou, X., Q. Ren, W. Li, et al. 2025. “Association Between Short‐Term Blood Pressure Variability and the Alzheimer's Disease Continuum.” Brain and Behavior 15, no. 10: e70990. 10.1002/brb3.70990

In the Funding section, the name of the funding program “Scientific and Technological Innovation 2030” was incorrect and should have read “Noncommunicable Chronic Diseases–National Science and Technology Major Project.” In addition, the funding source “Beijing Natural Science Foundation (Grant No. 7254343)” was missing. The updated Funding section is as follows:

**Funding**

This research was funded by Noncommunicable Chronic Diseases–National Science and Technology Major Project, National Key Research and Development Program of China, Beijing Natural Science Foundation, National Natural Science Foundation of China, and Key Project of Yunnan Provincial Department of Science and Technology (Grant Nos. 2023ZD0505804, 2024YFF0507500, 2021YFC2500100, 2021YFC2500103, 7254343, 82471212, 8207118, 82360387, 81870821, 202301AS070037, and 202305AK340001).

We apologize for these errors.

