# Supplementary figures and images for: Association Between Short‐Term Blood Pressure Variability and the Alzheimer's Disease Continuum
Source: Brain Behav. 2025 Oct 15;15(10):e70990. doi: 10.1002/brb3.70990 (PMC12528549; doi:10.1002/brb3.70990)

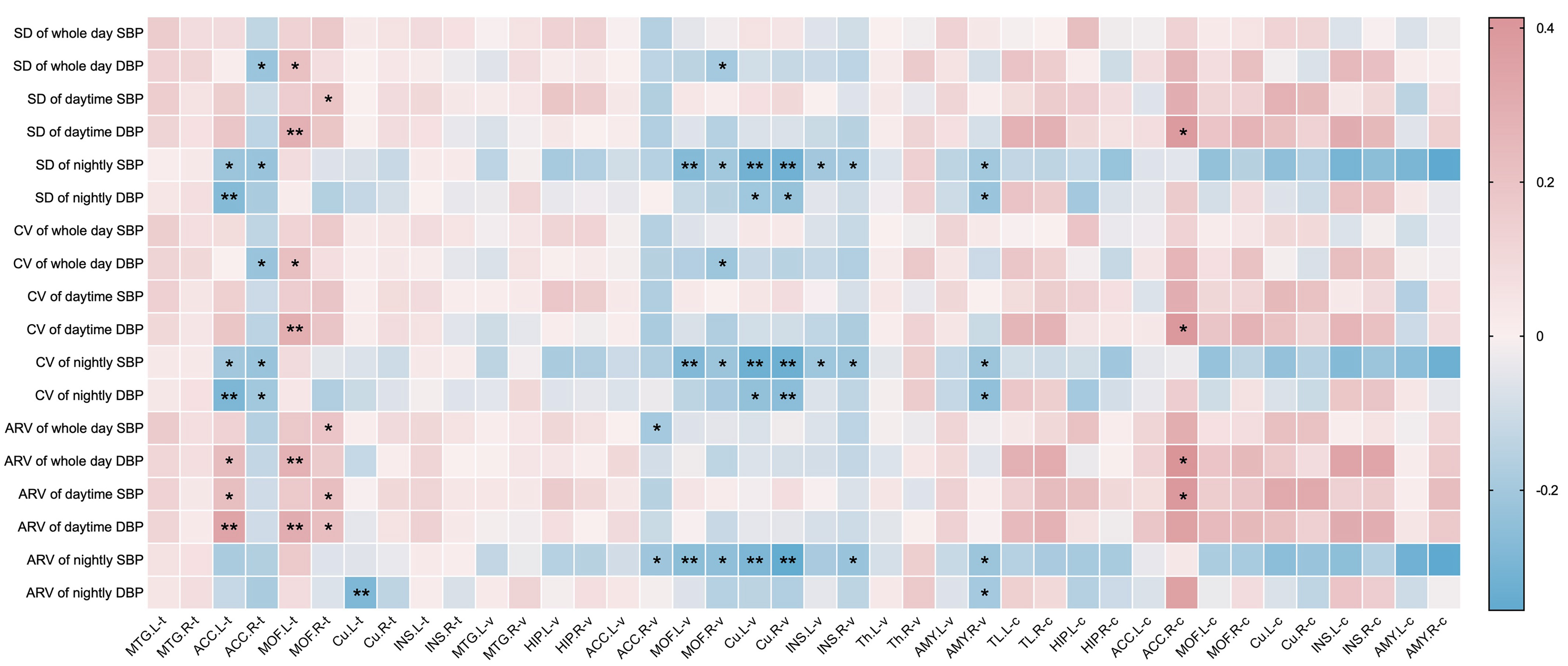

Supplement: Supplementary file 1 — Supplementary Figure: brb370990‐sup‐0001‐FigureS1.jpg [file BRB3-15-e70990-s001.jpg]
