# Supplementary material for: Association Between Short‐Term Blood Pressure Variability and the Alzheimer's Disease Continuum
Source: Brain Behav. 2025 Oct 15;15(10):e70990. doi: 10.1002/brb3.70990 (PMC12528549; doi:10.1002/brb3.70990)
Supplement: Supplementary file 2 — Supplementary Tables: brb370990‐sup‐0002‐TableS1‐S8.docx [file BRB3-15-e70990-s002.docx]

**Supplementary Figure 1 A heatmap of Partial Spearman’s correlation of BPV with multimodal neuroimaging measures across the AD continuum**

Age, sex, years of education, history of hypertension and coronary heart disease, and mean blood pressure were all adjusted to accurately assess the correlation

MTG: middle temporal gyrus; ACC: anterior cingulate cortex; MOF: medial orbitofrontal cortex; Cu: cuneus; INS: insula; HIP: hippocampus; Th: thalamus; AMY: amygdala; TL: temporal lobe; .L: left lateral brain area; .R: right lateral brain area; -t: thickness; -v: volume; -c: cerebral blood flow; SD: standard deviation; SBP: systolic blood pressure; DBP: diastolic blood pressure; CV: coefficient of variation; ARV: average real variability.

** indicates *P* < 0.01; * indicates *P* < 0.05.

**Table S1 The mediation of ROIs between SD of nightly SBP and HAMA scores across the AD continuum**

| Mediator | Path a  (SD of nightly SBP → ROIs) | | Path b  (ROIs → HAMA) | | Path a x b  (SD of nightly SBP →ROIs → HAMA) |
| --- | --- | --- | --- | --- | --- |
|  | 95% CIs | *P* | 95% CIs | *P* | 95% CIs |
| MTG.L-t | -0.029-0.025 | 0.890 | -2.797-4.353 | 0.662 | -0.102-0.032 |
| ACC.L-t | -0.036-0.006 | 0.158 | -2.826-6.289 | 0.447 | -0.163-0.057 |
| MOF.L-t | -0.025-0.019 | 0.785 | -4.122-4.913 | 0.861 | -0.054-0.068 |
| Cu.L-t | -0.030-0.004 | 0.130 | -5.089-6.301 | 0.831 | -0.084-0.062 |
| INS.L-t | -0.029-0.021 | 0.755 | -1.218-6.354 | 0.178 | -0.153-0.058 |
| MTG.L-v | -272.540-173.291 | 0.655 | -0.001-0.000 | 0.245 | -0.054-0.091 |
| HIP.L-v | -133.864-3.535 | 0.062 | -0.002-0.000 | 0.126 | -0.025-0.184 |
| ACC.L-v | -71.424-59.861 | 0.860 | -0.003-0.000 | 0.056 | -0.079-0.118 |
| MOF.L-v | -109.969-40.273 | 0.354 | -0.002-0.001 | 0.262 | -0.034-0.153 |
| Cu.L-v | -117.335-6.040 | 0.076 | -0.003- -0.000 | 0.030 | -0.012-0.225 |
| INS.L-v | -119.180-57.916 | 0.489 | -0.002-0.000 | 0.073 | -0.067-0.195 |
| Th.L-v | -84.025-132.711 | 0.652 | -0.002-0.000 | 0.028 | -0.139-0.094 |
| AMY.L-v | -41.278-18.200 | 0.438 | -0.004-0.003 | 0.696 | -0.104-0.055 |
| MTG.R-t | -0.030-0.018 | 0.611 | -4.196-4.068 | 0.975 | -0.115-0.052 |
| ACC.R-t | -0.051-0.001 | 0.055 | -0.789-6.567 | 0.120 | -0.291-0.081 |
| MOF.R-t | -0.038-0.005 | 0.137 | -6.234-2.825 | 0.451 | -0.084-0.158 |
| Cu.R-t | -0.038- -0.004 | 0.015 | -5.497-6.287 | 0.893 | -0.124-0.126 |
| INS.R-t | -0.024-0.024 | 0.973 | -2.094-5.979 | 0.336 | -0.123-0.119 |
| MTG.R-v | -216.092-217.019 | 0.997 | -0.001-0.000 | 0.084 | -0.100-0.100 |
| HIP.R-v | -125.085-11.263 | 0.099 | -0.003-0.000 | 0.106 | -0.049-0.206 |
| ACC.R-v | -84.607-27.397 | 0.308 | -0.003-0.000 | 0.056 | -0.042-0.182 |
| MOF.R-v | -94.801-25.695 | 0.253 | -0.003-0.000 | 0.095 | -0.026-0.186 |
| Cu.R-v | -118.028-12.548 | 0.110 | -0.002-0.001 | 0.200 | -0.043-0.162 |
| INS.R-v | -111.069-66.014 | 0.610 | -0.002- -0.000 | 0.024 | -0.091-0.242 |
| Th.R-v | -78.969-155.426 | 0.514 | -0.002-0.000 | 0.003 | -0.213-0.100 |
| AMY.R-v | -41.572-17.424 | 0.413 | -0.005-0.001 | 0.013 | -0.066-0.170 |

Age, sex, years of education, and history of hypertension and coronary heart disease were all adjusted to accurately assess the mediating effects.

ROI: region of interest; SD: standard deviation; SBP: systolic blood pressure; HAMA: Hamilton Anxiety Scale; AD: Alzheimer’s disease; CI: confidence interval; MTG: middle temporal gyrus; ACC: anterior cingulate cortex; MOF: medial orbitofrontal cortex; Cu: cuneus; INS: insula; HIP: hippocampus; Th: thalamus; AMY: amygdala; .L: left lateral brain area; .R: right lateral brain area; -t: thickness; -v: volume.

**Table S2 The mediation of ROIs between CV of nightly SBP and HAMA scores across the AD continuum**

| Mediator | Path a  (CV of nightly SBP → ROIs) | | Path b  (ROIs → HAMA) | | Path a x b  (CV of nightly SBP →ROIs → HAMA) |
| --- | --- | --- | --- | --- | --- |
|  | 95% CIs | *P* | 95% CIs | *P* | 95% CIs |
| MTG.L-t | -0.037-0.027 | 0.743 | -2.721-4.533 | 0.616 | -0.116-0.035 |
| ACC.L-t | -0.042-0.008 | 0.172 | -3.045-6.198 | 0.494 | -0.155-0.076 |
| MOF.L-t | -0.034-0.016 | 0.463 | -3.809-5.398 | 0.729 | -0.089-0.079 |
| Cu.L-t | -0.037-0.003 | 0.091 | -5.150-6.485 | 0.818 | -0.108-0.084 |
| INS.L-t | -0.032-0.027 | 0.874 | -1.424-6.274 | 0.210 | -0.130-0.081 |
| MTG.L-v | -386.025-126.668 | 0.313 | -0.001-0.000 | 0.344 | -0.031-0.128 |
| HIP.L-v | -161.698- -3.439 | 0.041 | -0.003-0.000 | 0.129 | -0.021-0.242 |
| ACC.L-v | -91.813-60.467 | 0.680 | -0.003-0.000 | 0.073 | -0.065-0.154 |
| MOF.L-v | -138.902-34.394 | 0.230 | -0.002-0.001 | 0.300 | -0.055-0.193 |
| Cu.L-v | -149.428- -9.096 | 0.028 | -0.003- -0.000 | 0.038 | 0.002-0.301 |
| INS.L-v | -147.940-57.044 | 0.376 | -0.002-0.000 | 0.086 | -0.061-0.263 |
| Th.L-v | -106.924-145.267 | 0.760 | -0.002- -0.000 | 0.037 | -0.152-0.159 |
| AMY.L-v | -52.938-15.677 | 0.279 | -0.004-0.003 | 0.769 | -0.111-0.079 |
| MTG.R-t | -0.040-0.015 | 0.350 | -3.971-4.475 | 0.905 | -0.134-0.067 |
| ACC.R-t | -0.062- -0.003 | 0.030 | -0.753-6.793 | 0.114 | -0.318-0.077 |
| MOF.R-t | -0.045-0.004 | 0.101 | -6.328-2.919 | 0.460 | -0.092-0.205 |
| Cu.R-t | -0.043- -0.004 | 0.018 | -5.883-6.023 | 0.981 | -0.160-0.146 |
| INS.R-t | -0.031-0.025 | 0.826 | -1.935-6.238 | 0.293 | -0.160-0.119 |
| MTG.R-v | -287.139-215.601 | 0.775 | -0.000-0.000 | 0.113 | -0.084-0.141 |
| HIP.R-v | -158.767- -3.176 | 0.042 | -0.003-0.000 | 0.128 | -0.044-0.283 |
| ACC.R-v | -92.081-38.677 | 0.414 | -0.003-0.000 | 0.046 | -0.054-0.216 |
| MOF.R-v | -107.055-33.326 | 0.295 | -0.003-0.000 | 0.086 | -0.039-0.201 |
| Cu.R-v | -132.173-20.400 | 0.147 | -0.003-0.001 | 0.171 | -0.060-0.192 |
| INS.R-v | -150.973-53.086 | 0.338 | -0.002- -0.000 | 0.039 | -0.071-0.318 |
| Th.R-v | -100.206- 172.648 | 0.595 | -0.002- -0.000 | 0.005 | -0.215-0.134 |
| AMY.R-v | -54.179-13.738 | 0.236 | -0.005-0.002 | 0.293 | -0.047-0.227 |

Age, sex, years of education, and history of hypertension and coronary heart disease were all adjusted to accurately assess the mediating effects.

ROI: region of interest; CV: coefficient of variation; SBP: systolic blood pressure; HAMA: Hamilton Anxiety Scale; AD: Alzheimer’s disease; CI: confidence interval; MTG: middle temporal gyrus; ACC: anterior cingulate cortex; MOF: medial orbitofrontal cortex; Cu: cuneus; INS: insula; HIP: hippocampus; Th: thalamus; AMY: amygdala; .L: left lateral brain area; .R: right lateral brain area; -t: thickness; -v: volume.

**Table S3 The mediation of ROIs between SD of nightly DBP and NPI scores across the AD continuum**

| Mediator | Path a  (SD of nightly DBP → ROIs) | | Path b  (ROIs → NPI) | | Path a x b  (SD of nightly SBP →ROIs → NPI) |
| --- | --- | --- | --- | --- | --- |
|  | 95% CIs | *P* | 95% CIs | *P* | 95% CIs |
| MTG.L-t | -0.021-0.023 | 0.946 | -29.139- -1.959 | 0.026 | -0.391-0.463 |
| ACC.L-t | -0.042- -0.003 | 0.025 | -28.461-3.350 | 0.119 | -0.049-0.718 |
| MOF.L-t | -0.028-0.011 | 0.366 | -26.651-5.807 | 0.202 | -0.122-0.459 |
| Cu.L-t | -0.025-0.006 | 0.220 | -32.160-8.747 | 0.255 | -0.101-0.425 |
| INS.L-t | -0.034-0.009 | 0.249 | -20.293-8.772 | 0.429 | -0.212-0.341 |
| MTG.L-v | -267.948-114.917 | 0.425 | -0.003-0.000 | 0.115 | -0.153-0.586 |
| HIP.L-v | -67.831-49.967 | 0.761 | -0.012- -0.002 | 0.012 | -0.321-0.456 |
| ACC.L-v | -96.677-16.165 | 0.158 | -0.008-0.003 | 0.431 | -0.167-0.433 |
| MOF.L-v | -79.522-45.230 | 0.582 | -0.010- -0.000 | 0.033 | -0.283-0.539 |
| Cu.L-v | -87.329-34.412 | 0.386 | -0.006-0.005 | 0.901 | -0.182-0.221 |
| INS.L-v | -108.347-48.516 | 0.446 | -0.007-0.001 | 0.141 | -0.218-0.438 |
| Th.L-v | -79.954-126.991 | 0.649 | -0.005-0.001 | 0.289 | -0.516-0.153 |
| AMY.L-v | -35.177-14.995 | 0.422 | -0.024-0.000 | 0.057 | -0.189-0.551 |
| MTG.R-t | -0.011-0.027 | 0.389 | -42.025- -13.604 | <0.001 | -0.956-0.355 |
| ACC.R-t | -0.035-0.014 | 0.395 | -23.116-1.604 | 0.086 | -0.175-0.468 |
| MOF.R-t | -0.036-0.000 | 0.055 | -36.590- -3.915 | 0.016 | -0.010-0.905 |
| Cu.R-t | -0.023-0.007 | 0.310 | -39.710-0.363 | 0.054 | -0.236-0.536 |
| INS.R-t | -0.027-0.015 | 0.554 | -29.096- -0.507 | 0.043 | -0.249-0.538 |
| MTG.R-v | -102.126-262.685 | 0.380 | -0.004- -0.001 | 0.001 | -0.954-0.335 |
| HIP.R-v | -41.418-85.397 | 0.488 | -0.007-0.003 | 0.399 | -0.448-0.149 |
| ACC.R-v | -61.679-35.491 | 0.590 | -0.014- -0.001 | 0.019 | -0.324-0.624 |
| MOF.R-v | -86.646-15.155 | 0.164 | -0.014- -0.003 | 0.004 | -0.167-0.934 |
| Cu.R-v | -67.112-52.362 | 0.805 | -0.009-0.001 | 0.149 | -0.308-0.271 |
| INS.R-v | -105.572-44.852 | 0.420 | -0.009- -0.001 | 0.020 | -0.319-0.641 |
| Th.R-v | -73.504-140.771 | 0.530 | -0.004-0.002 | 0.316 | -0.560-0.102 |
| AMY.R-v | -40.522-13.569 | 0.321 | -0.023- -0.001 | 0.034 | -0.231-0.660 |

Age, sex, years of education, and history of hypertension and coronary heart disease were all adjusted to accurately assess the mediating effects.

ROI: region of interest; SD: standard deviation; DBP: diastolic blood pressure; NPI: Neuropsychiatric Inventory; AD: Alzheimer’s disease; CI: confidence interval; MTG: middle temporal gyrus; ACC: anterior cingulate cortex; MOF: medial orbitofrontal cortex; Cu: cuneus; INS: insula; HIP: hippocampus; Th: thalamus; AMY: amygdala; .L: left lateral brain area; .R: right lateral brain area; -t: thickness; -v: volume.

**Table S4 The mediation of ROIs between CV of nightly DBP and NPI scores across the AD continuum**

| Mediator | Path a  (CV of nightly DBP → ROIs) | | Path b  (ROIs → NPI) | | Path a x b  (CV of nightly SBP →ROIs → NPI) |
| --- | --- | --- | --- | --- | --- |
|  | 95% CIs | *P* | 95% CIs | *P* | 95% CIs |
| MTG.L-t | -0.018-0.013 | 0.757 | -28.517- -0.917 | 0.037 | -0.193-0.361 |
| ACC.L-t | -0.029- -0.003 | 0.019 | -28.933-3.260 | 0.115 | -0.028-0.521 |
| MOF.L-t | -0.020-0.006 | 0.299 | -26.704-6.107 | 0.212 | -0.058-0.327 |
| Cu.L-t | -0.020-0.001 | 0.073 | -31.662-10.494 | 0.317 | -0.046-0.339 |
| INS.L-t | -0.024-0.005 | 0.205 | -20.401-8.969 | 0.437 | -0.157-0.236 |
| MTG.L-v | -208.807-53.175 | 0.238 | -0.003-0.000 | 0.141 | -0.062-0.454 |
| HIP.L-v | -48.879-32.363 | 0.684 | -0.012- -0.001 | 0.014 | -0.202-0.352 |
| ACC.L-v | -66.760-11.125 | 0.157 | -0.008-0.003 | 0.416 | -0.152-0.266 |
| MOF.L-v | -54.313-31.827 | 0.602 | -0.010- -0.001 | 0.033 | -0.167-0.357 |
| Cu.L-v | -60.704-23.292 | 0.374 | -0.006-0.005 | 0.892 | -0.142-0.159 |
| INS.L-v | -78.431-29.552 | 0.367 | -0.007-0.001 | 0.150 | -0.179-0.316 |
| Th.L-v | -37.836-103.934 | 0.353 | -0.005-0.001 | 0.229 | -0.383-0.112 |
| AMY.L-v | -28.410-5.796 | 0.190 | -0.024-0.001 | 0.078 | -0.078-0.459 |
| MTG.R-t | -0.011-2.528 | <0.001 | -40.386- -11.284 | 0.001 | -0.420-0.358 |
| ACC.R-t | -0.029-0.005 | 0.162 | -22.767-2.623 | 0.117 | -0.066-0.366 |
| MOF.R-t | -0.025- -0.000 | 0.044 | -37.053- -4.024 | 0.016 | 0.011-0.657 |
| Cu.R-t | -0.018-0.003 | 0.136 | -39.434-1.698 | 0.071 | -0.068-0.456 |
| INS.R-t | -0.020-0.009 | 0.449 | -29.047- -0.113 | 0.048 | -0.128-0.385 |
| MTG.R-v | -72.343-179.611 | 0.396 | -0.004- -0.001 | 0.001 | -0.585-0.262 |
| HIP.R-v | -29.389-58.193 | 0.511 | -0.007-0.003 | 0.417 | -0.263-0.121 |
| ACC.R-v | -36.899-30.368 | 0.846 | -0.014- -0.002 | 0.014 | -0.236-0.410 |
| MOF.R-v | -57.660-12.891 | 0.208 | -0.014- -0.003 | 0.004 | -0.094-0.604 |
| Cu.R-v | -44.064-38.444 | 0.891 | -0.009-0.001 | 0.140 | -0.230-0.202 |
| INS.R-v | -75.878-27.701 | 0.354 | -0.009- -0.001 | 0.022 | -0.192-0.468 |
| Th.R-v | -29.938-116.302 | 0.240 | -0.005-0.001 | 0.242 | -0.373-0.059 |
| AMY.R-v | -31.227-5.732 | 0.172 | -0.023- -0.000 | 0.043 | -0.086-0.558 |

Age, sex, years of education, and history of hypertension and coronary heart disease were all adjusted to accurately assess the mediating effects.

ROI: region of interest; CV: coefficient of variation; DBP: diastolic blood pressure; NPI: Neuropsychiatric Inventory; AD: Alzheimer’s disease; CI: confidence interval; MTG: middle temporal gyrus; ACC: anterior cingulate cortex; MOF: medial orbitofrontal cortex; Cu: cuneus; INS: insula; HIP: hippocampus; Th: thalamus; AMY: amygdala; .L: left lateral brain area; .R: right lateral brain area; -t: thickness; -v: volume.

**Table S5 The mediation of ROIs between ARV of nightly DBP and NPI scores across the AD continuum**

| Mediator | Path a  (ARV of nightly DBP → ROIs) | | Path b  (ROIs → NPI) | | Path a x b  (ARV of nightly DBP →ROIs → NPI) |
| --- | --- | --- | --- | --- | --- |
|  | 95% CIs | *P* | 95% CIs | *P* | 95% CIs |
| MTG.L-t | -0.019-0.010 | 0.555 | -28.120- -0.266 | 0.046 | -0.136-0.326 |
| ACC.L-t | -0.028- -0.001 | 0.031 | -29.110-2.757 | 0.103 | -0.013-0.552 |
| MOF.L-t | -0.016-0.010 | 0.627 | -27.736-4.658 | 0.158 | -0.131-0.247 |
| Cu.L-t | -0.024- -0.005 | 0.003 | -30.735-14.656 | 0.479 | -0.150-0.403 |
| INS.L-t | -0.025-0.004 | 0.151 | -20.261-9.345 | 0.461 | -0.116-0.234 |
| MTG.L-v | -175.365-82.932 | 0.475 | -0.003-0.000 | 0.108 | -0.091-0.325 |
| HIP.L-v | -35.084-44.308 | 0.816 | -0.012- -0.002 | 0.008 | -0.333-0.257 |
| ACC.L-v | -57.439-19.486 | 0.326 | -0.008-0.003 | 0.346 | -0.109-0.225 |
| MOF.L-v | -48.435-35.812 | 0.764 | -0.010- -0.001 | 0.028 | -0.245-0.264 |
| Cu.L-v | -36.427-46.245 | 0.812 | -0.007-0.004 | 0.629 | -0.110-0.132 |
| INS.L-v | -72.981-32.694 | 0.446 | -0.007-0.001 | 0.138 | -0.172-0.256 |
| Th.L-v | -18.149-118.246 | 0.146 | -0.005-0.001 | 0.169 | -0.453-0.108 |
| AMY.L-v | -24.276-9.477 | 0.382 | -0.024-0.001 | 0.059 | -0.102-0.355 |
| MTG.R-t | -0.013-0.013 | 0.957 | -39.577- -10.114 | 0.002 | -0.439-0.377 |
| ACC.R-t | -0.035- -0.004 | 0.016 | -22.362-4.453 | 0.185 | -0.047-0.526 |
| MOF.R-t | -0.021-0.004 | 0.185 | -37.307- -5.394 | 0.010 | -0.118-0.529 |
| Cu.R-t | -0.018-0.003 | 0.143 | -39.601-1.553 | 0.069 | -0.089-0.421 |
| INS.R-t | -0.023-0.005 | 0.222 | -28.580-0.891 | 0.065 | -0.098-0.407 |
| MTG.R-v | -93.280-153.983 | 0.623 | -0.004- -0.001 | 0.002 | -0.560-0.230 |
| HIP.R-v | -0.494-81.783 | 0.053 | -0.008-0.002 | 0.215 | -0.519-0.147 |
| ACC.R-v | -49.416-15.448 | 0.297 | -0.013- -0.001 | 0.028 | -0.145-0.476 |
| MOF.R-v | -63.054-4.800 | 0.091 | -0.014- -0.003 | 0.005 | -0.074-0.594 |
| Cu.R-v | -30.707-49.630 | 0.637 | -0.010-0.001 | 0.095 | -0.367-0.195 |
| INS.R-v | -78.420-22.238 | 0.267 | -0.009- -0.001 | 0.025 | -0.234-0.405 |
| Th.R-v | 6.050-143.730 | 0.034 | -0.005-0.001 | 0.133 | -0.581-0.056 |
| AMY.R-v | -33.587-1.997 | 0.081 | -0.023-0.000 | 0.053 | -0.068-0.571 |

Age, sex, years of education, and history of hypertension and coronary heart disease were all adjusted to accurately assess the mediating effects.

ROI: region of interest; ARV: average real variability; DBP: diastolic blood pressure; NPI: Neuropsychiatric Inventory; AD: Alzheimer’s disease; CI: confidence interval; MTG: middle temporal gyrus; ACC: anterior cingulate cortex; MOF: medial orbitofrontal cortex; Cu: cuneus; INS: insula; HIP: hippocampus; Th: thalamus; AMY: amygdala; .L: left lateral brain area; .R: right lateral brain area; -t: thickness; -v: volume.

**Table S6 The mediation of ROIs between ARV of whole day SBP and HAMD scores across the AD continuum**

| Mediator | Path a  (ARV of whole day SBP → ROIs) | | Path b  (ROIs → HAMD) | | Path a x b  (ARV of whole day SBP →ROIs → HAMD) |
| --- | --- | --- | --- | --- | --- |
|  | 95% CIs | *P* | 95% CIs | *P* | 95% CIs |
| MTG.L-t | -0.015-0.032 | 0.490 | -7.529-3.357 | 0.443 | -0.153-0.056 |
| ACC.L-t | -0.016-0.022 | 0.736 | -8.209-5.406 | 0.679 | -0.097-0097 |
| MOF.L-t | -0.012-0.026 | 0.454 | -8.142-5.673 | 0.720 | -0.083-0.069 |
| Cu.L-t | -0.024-0.006 | 0.251 | -13.927-2.820 | 0.188 | -0.047-0.230 |
| INS.L-t | -0.015-0.028 | 0.558 | -4.640-7.165 | 0.668 | -0.077-0.097 |
| MTG.L-v | -187.236-199.987 | 0.947 | -0.001-0.000 | 0.078 | -0.152-0.128 |
| HIP.L-v | -53.012-71.243 | 0.768 | -0.004-0.000 | 0.084 | -0.142-0.142 |
| ACC.L-v | -35.268-77.711 | 0.452 | -0.004-0.001 | 0.125 | -0.171-0.038 |
| MOF.L-v | -69.573-61.997 | 0.908 | -0.002-0.002 | 0.623 | -0.064-0.099 |
| Cu.L-v | -77.864-32.430 | 0.410 | -0.004-0.000 | 0.105 | -0.058-0.244 |
| INS.L-v | -102.774-50.696 | 0.497 | -0.003-0.001 | 0.151 | -0.079-0.183 |
| Th.L-v | -100.483-87.737 | 0.892 | -0.002-0.001 | 0.393 | -0.086-0.113 |
| AMY.L-v | -19.229-32.524 | 0.607 | -0.008-0.002 | 0.186 | -0.141-0.087 |
| MTG.R-t | -0.021-0.021 | 0.998 | -8.077-4.399 | 0.554 | -0.110-0.116 |
| ACC.R-t | -0.030-0.017 | 0.576 | -5.390-5.674 | 0.959 | -0.091-0.238 |
| MOF.R-t | -0.017-0.022 | 0.785 | -11.128-2.067 | 0.173 | -0.114-0.167 |
| Cu.R-t | -0.026-0.004 | 0.158 | -10.904-6.087 | 0.570 | -0.122-0.194 |
| INS.R-t | -0.002-0.038 | 0.080 | -6.989-5.928 | 0.869 | -0.147-0.179 |
| MTG.R-v | -284.596-85.201 | 0.282 | -0.001-0.000 | 0.262 | -0.042-0.190 |
| HIP.R-v | -49.209-72.820 | 0.698 | -0.004-0.001 | 0.149 | -0.155-0.153 |
| ACC.R-v | -77.992-18.475 | 0.220 | -0.004-0.002 | 0.432 | -0.041-0.170 |
| MOF.R-v | -82.013-22.400 | 0.255 | -0.004-0.001 | 0.133 | -0.041-0.242 |
| Cu.R-v | -89.888-25.103 | 0.262 | -0.003-0.002 | 0.795 | -0.081-0.203 |
| INS.R-v | -110.675-41.668 | 0.365 | -0.003-0.000 | 0.055 | -0.067-0.250 |
| Th.R-v | -133.395-69.770 | 0.530 | -0.002-0.001 | 0.225 | -0.072-0.215 |
| AMY.R-v | -27.599-23.937 | 0.887 | -0.008-0.001 | 0.145 | -0.100-0.161 |

Age, sex, years of education, and history of hypertension and coronary heart disease were all adjusted to accurately assess the mediating effects.

ROI: region of interest; ARV: average real variability; SBP: systolic blood pressure; HAMD: Hamilton Depression Scale; AD: Alzheimer’s disease; CI: confidence interval; MTG: middle temporal gyrus; ACC: anterior cingulate cortex; MOF: medial orbitofrontal cortex; Cu: cuneus; INS: insula; HIP: hippocampus; Th: thalamus; AMY: amygdala; .L: left lateral brain area; .R: right lateral brain area; -t: thickness; -v: volume.

**Table S7 The mediation of ROIs between ARV of nightly SBP and HAMD scores across the AD continuum**

| Mediator | Path a  (ARV of nightly SBP → ROIs) | | Path b  (ROIs → HAMD) | | Path a x b  (ARV of nightly SBP →ROIs → HAMD) |
| --- | --- | --- | --- | --- | --- |
|  | 95% CIs | *P* | 95% CIs | *P* | 95% CIs |
| MTG.L-t | -0.016-0.014 | 0.908 | -6.358-3.747 | 0.604 | -0.061-0.035 |
| ACC.L-t | -0.016-0.009 | 0.481 | -6.244-6.505 | 0.967 | -0.082-0.066 |
| MOF.L-t | -0.010-0.014 | 0.735 | -7.348-5.432 | 0.763 | -0.056-0.065 |
| Cu.L-t | -0.022- -0.004 | 0.006 | -10.499-6.690 | 0.657 | -0.079-0.145 |
| INS.L-t | -0.015-0.013 | 0.886 | -3.429-7.451 | 0.459 | -0.059-0.039 |
| MTG.L-v | -175.194-72.336 | 0.406 | -0.001-0.000 | 0.152 | -0.046-0.100 |
| HIP.L-v | -73.344-3.718 | 0.075 | -0.003-0.001 | 0.398 | -0.047-0.116 |
| ACC.L-v | -48.662-24.298 | 0.504 | -0.003-0.001 | 0.308 | -0.035-0.069 |
| MOF.L-v | -51.153-33.513 | 0.676 | -0.002-0.001 | 0.725 | -0.040-0.085 |
| Cu.L-v | -58.849-11.253 | 0.178 | -0.004-0.001 | 0.183 | -0.029-0.159 |
| INS.L-v | -79.903-17.628 | 0.204 | -0.002-0.001 | 0261 | -0.031-0.132 |
| Th.L-v | -59.838-61.557 | 0.977 | -0.002-0.001 | 0.326 | -0.076-0.067 |
| AMY.L-v | -27.617-5.082 | 0.171 | -0.006-0.004 | 0.595 | -0.078-0.076 |
| MTG.R-t | -0.019-0.008 | 0.396 | -6.448-5.306 | 0.845 | -0.059-0.074 |
| ACC.R-t | -0.023-0.007 | 0.286 | -4.096-6.276 | 0.673 | -0.251-0.076 |
| MOF.R-t | -0.020-0.004 | 0.187 | -8.569-4.188 | 0.491 | -0.053-0.145 |
| Cu.R-t | -0.021- -0.002 | 0.018 | -7.784-8.822 | 0.900 | -0.102-0.113 |
| INS.R-t | -0.014-0.013 | 0.913 | -4.376-7.149 | 0.629 | -0.076-0.070 |
| MTG.R-v | -191.967-45.425 | 0.219 | -0.001-0.000 | 0.349 | -0.025-0.126 |
| HIP.R-v | -66.117-10.706 | 0.153 | -0.003-0.001 | 0.499 | -0.057-0.104 |
| ACC.R-v | -59.014-1.683 | 0.064 | -0.003-0.002 | 0.755 | -0.052-0.104 |
| MOF.R-v | -62.523-3.242 | 0.076 | -0.004-0.001 | 0.272 | -0.031-0.175 |
| Cu.R-v | -56.269-18.077 | 0.305 | -0.002-0.002 | 0.896 | -0.060-0.100 |
| INS.R-v | -75.528-22.259 | 0.278 | -0.003-0.000 | 0.071 | -0.059-0.174 |
| Th.R-v | -54.711-76.760 | 0.736 | -0.002-0.000 | 0.086 | -0.137-0.085 |
| AMY.R-v | -32.972- -1.623 | 0.031 | -0.006-0.004 | 0.594 | -0.077-0.128 |

Age, sex, years of education, and history of hypertension and coronary heart disease were all adjusted to accurately assess the mediating effects.

ROI: region of interest; ARV: average real variability; SBP: systolic blood pressure; HAMD: Hamilton Depression Scale; AD: Alzheimer’s disease; CI: confidence interval; MTG: middle temporal gyrus; ACC: anterior cingulate cortex; MOF: medial orbitofrontal cortex; Cu: cuneus; INS: insula; HIP: hippocampus; Th: thalamus; AMY: amygdala; .L: left lateral brain area; .R: right lateral brain area; -t: thickness; -v: volume.

**Table S8 The mediation of ROIs between ARV of nightly SBP and HAMA scores across the AD continuum**

| Mediator | Path a  (ARV of nightly SBP → ROIs) | | Path b  (ROIs → HAMA) | | Path a x b  (ARV of nightly SBP →ROIs → HAMA) |
| --- | --- | --- | --- | --- | --- |
|  | 95% CIs | *P* | 95% CIs | *P* | 95% CIs |
| MTG.L-t | -0.016-0.014 | 0.908 | -2.469-4.057 | 0.625 | -0.041-0.020 |
| ACC.L-t | -0.016-0.008 | 0.481 | -2.849-5.341 | 0.542 | -0.059-0.031 |
| MOF.L-t | -0.010-0.014 | 0.735 | -4.448-3.809 | 0.877 | -0.023-0.048 |
| Cu.L-t | -0.022- -0.004 | 0.006 | -1.731-9.132 | 0.176 | -0.131-0.006 |
| INS.L-t | -0.015-0.013 | 0.886 | -0.979-5.912 | 0.156 | -0.054-0.037 |
| MTG.L-v | -175.194-72.336 | 0.406 | -0.001-0.000 | 0.343 | -0.024-0.054 |
| HIP.L-v | -73.344-3.718 | 0.075 | -0.002-0.000 | 0.166 | -0.034-0.081 |
| ACC.L-v | -48.662-24.298 | 0.504 | -0.003-0.000 | 0.082 | -0.024-0.064 |
| MOF.L-v | -51.153-33.513 | 0.676 | -0.002-0.000 | 0.164 | -0.028-0.080 |
| Cu.L-v | -58.849-11.253 | 0.178 | -0.003- -0.000 | 0.021 | -0.038-0.106 |
| INS.L-v | -79.903-17.628 | 0.204 | -0.002-0.000 | 0.140 | -0.025-0.101 |
| Th.L-v | -59.838-61.557 | 0.977 | -0.002- -0.000 | 0.028 | -0.089-0.057 |
| AMY.L-v | -27.617-5.082 | 0.171 | -0.003-0.003 | 0.932 | -0.069-0.031 |
| MTG.R-t | -0.019-0.008 | 0.396 | -3.274-4.311 | 0.784 | -0.056-0.030 |
| ACC.R-t | -0.023-0.007 | 0.286 | -0.901-5.633 | 0.151 | -0.218-0.057 |
| MOF.R-t | -0.020-0.004 | 0.187 | -5.487-2.752 | 0.506 | -0.043-0.081 |
| Cu.R-t | -0.021- -0.002 | 0.018 | -3.892-6.789 | 0.586 | -0.085-0.048 |
| INS.R-t | -0.014-0.013 | 0.913 | -1.566-5.772 | 0.253 | -0.059-0.061 |
| MTG.R-v | -191.967-45.425 | 0.219 | -0.001-0.000 | 0.273 | -0.016-0.074 |
| HIP.R-v | -66.117-10.706 | 0.153 | -0.002-0.000 | 0.109 | -0.043-0.085 |
| ACC.R-v | -59.014-1.683 | 0.064 | -0.003-0.001 | 0.162 | -0.007-0.098 |
| MOF.R-v | -62.523-3.242 | 0.076 | -0.002-0.001 | 0.217 | -0.024-0.106 |
| Cu.R-v | -56.269-18.077 | 0.305 | -0.002-0.000 | 0.142 | -0.070-0.070 |
| INS.R-v | -75.528-22.259 | 0.278 | -0.002-0.000 | 0.045 | -0.042-0.125 |
| Th.R-v | -54.711-76.760 | 0.736 | -0.002- -0.000 | 0.002 | -0.147-0.059 |
| AMY.R-v | -32.972- -1.623 | 0.031 | -0.004-0.003 | 0.749 | -0.059-0.076 |

Age, sex, years of education, and history of hypertension and coronary heart disease were all adjusted to accurately assess the mediating effects.

ROI: region of interest; ARV: average real variability; SBP: systolic blood pressure; HAMA: Hamilton Anxiety Scale; AD: Alzheimer’s disease; CI: confidence interval; MTG: middle temporal gyrus; ACC: anterior cingulate cortex; MOF: medial orbitofrontal cortex; Cu: cuneus; INS: insula; HIP: hippocampus; Th: thalamus; AMY: amygdala; .L: left lateral brain area; .R: right lateral brain area; -t: thickness; -v: volume.
